# Oral Health-Related Quality of Life, Behaviours and Oral Manifestations in a Paediatric Population with Type I Diabetes Mellitus: A Comparative Cross-Sectional Study

**DOI:** 10.3390/dj13090425

**Published:** 2025-09-15

**Authors:** Patrícia João, Ana Coelho Canta, Sónia Mendes

**Affiliations:** Faculdade de Medicina Dentária, Universidade de Lisboa, Rua Professora Teresa Ambrósio, 1600-277 Lisbon, Portugalsoniaborralho@edu.ulisboa.pt (S.M.)

**Keywords:** diabetes mellitus, type 1, behaviour, oral health, oral manifestations, quality of life, child health

## Abstract

**Introduction:** Type 1 diabetes mellitus (DM1) can influence oral health, increasing susceptibility to various oral manifestations. **Objectives:** This study aimed to characterise oral health-related quality of life (OHRQoL), reported oral symptoms, and oral health behaviours in paediatric individuals with DM1, and compare them with those without DM1; as well as to identify factors associated with OHRQoL in both groups. **Methods:** A cross-sectional study was conducted using an online questionnaire completed by caregivers of Portuguese children with or without DM1. The questionnaire collected information on sociodemographic, oral health behaviours, perceived oral health, reported oral manifestations, and OHRQoL using the Early Childhood Oral Health Impact Scale (ECOHIS). Descriptive statistics were calculated, along with chi-square tests and negative binomial regression analyses (α = 0.05). **Results:** The sample included 235 individuals (115 with DM1 and 120 without). No significant differences were observed in overall OHRQoL between the groups, although children with DM1 had slightly higher ECOHIS total scores (4.38 vs. 4.02). Oral health behaviours were comparable in both groups. Children with DM1 reported significantly more xerostomia (*p* < 0.001). Within the DM1 group, better OHRQoL was significantly related to the following variables: female sex, absence of caries, absence of gingivitis, and no taste changes. In contrast, in the non-DM1 group, OHRQoL was associated with different conditions: the caregiver perception, frequent sugar intake, recurrent aphthous stomatitis, burning mouth sensations, and age. **Conclusion:** Although OHRQoL levels and oral health behaviours were similar between children with and without DM1, the factors influencing these outcomes varied considerably, emphasising the need for targeted oral health strategies tailored to the specific context of this chronic condition.

## 1. Introduction

Diabetes mellitus (DM) is a chronic metabolic disease characterised by defects in the secretion or function of insulin, the hormone responsible for maintaining stable blood glucose levels. The primary symptoms of this condition are caused by hyperglycaemia and include polyuria, polydipsia, weight loss, blurred vision, and constant tiredness, among others [1,2]. This hyperglycaemia state can affect the patient’s health, with the most common complications being an increased risk of cardiovascular disease and stroke, neuropathy, nephropathy, and retinopathy [3,4,5]. Moreover, diabetes can negatively impact oral health through mechanisms such as hyposalivation and immune suppression. Reduced salivary flow can impair the natural cleansing of the oral cavity, resulting in difficulties with chewing, swallowing, and maintaining oral comfort. In addition, impaired immune function compromises the body’s ability to fight infections and repair tissues, resulting in delayed wound healing, increased susceptibility to periodontal disease, and a higher risk of opportunistic oral infections, such as candidiasis. Overall, these factors may also contribute to greater accumulation of oral biofilm, which in turn exacerbates gingival inflammation and caries risk, creating a cycle that further deteriorates oral health. These patients might experience difficulties in healing, increased susceptibility to oral infections, or greater accumulation of oral biofilm [6,7].

Type I DM (DM1) results from an autoimmune response against the beta cells in the pancreas, leading to an insulin deficiency that affects carbohydrate metabolism [1,8]. The exact cause of DM1 remains unknown, although some studies have associated it with genetic factors, such as HLA proteins, and non-genetic factors, including specific viral infections [1].

The 11th edition of the “IDF Diabetes Atlas” by the International Diabetes Federation estimated that, in 2024, among people aged 20–79 years, 589 million globally had some form of DM, with 1.81 million individuals under 20 years old having DM1. Europe is the most affected region worldwide, followed by North America and the Caribbean [9]. In Portugal, according to the 2023 National Diabetes Programme, there were 1497 registered cases of DM1 in children aged 14 years and under, and 2982 cases when including those aged 19 years or less [10].

Although the association between diabetes and oral health is well documented, most studies have focused on adult populations, with relatively few investigations in children and adolescents. Particularly, the relationship between DM1 and Oral Health-Related Quality of Life (OHRQoL) in paediatric populations remains less studied, and this is particularly true in the Portuguese population. Furthermore, previous research has focused on objective clinical indicators. At the same time, subjective measures, which capture the impact of oral conditions on self-esteem, social interactions, eating habits, and emotional well-being, have consistently received less attention. Considering that children and adolescents are at critical stages of physical and psychosocial development, understanding these subjective impacts may be relevant.

Recently, a meta-analysis and systematic review conducted by Homagarani et al. [11] suggested that there was no statistically significant association between diabetes and OHRQoL. However, diabetes can negatively impact patients’ well-being by impairing oral health and increasing the risk of developing certain oral conditions, such as xerostomia, periodontal disease, and post-surgical infections, among others [11,12].

OHRQoL is a complex multidimensional concept that characterises an individual’s perception of how oral health influences their quality of life and overall well-being. In this way, OHRQoL focuses on the impact that oral health has on various aspects of the individual, namely their physical, psychological, functional, and social dimensions, reflecting comfort when performing daily activities such as eating, sleeping, and social interaction, as well as their self-esteem and satisfaction with their oral health [13].

In paediatric individuals, the OHRQoL is even more difficult to understand as it reflects not only the impact that oral health has on children’s lives but also that of their caregivers. Establishing good oral hygiene habits in children can be challenging, leading to oral health issues that cause parents to feel guilty and concerned. Moreover, the necessity of taking children to the dentist may require parents to miss work, and together with the costs of treatments, this can result in a decline in family income, impose a financial burden, and potentially impair their overall quality of life [14].

Considering the reasons mentioned, this study contributes to the integration of both reported diseases and symptom-related variables (such as oral pain, caries, gingival status, and taste changes), behavioural factors (such as dietary and oral hygiene habits), and caregiver perceptions. This integration provides a comprehensive understanding of how biological and psychosocial domains interact to shape OHRQoL outcomes. This study aims to provide original data on the OHRQoL of a Portuguese paediatric population with and without DM1. In this way, it wants to contribute to a broader understanding of the oral implications of the disease and to support the development of preventive strategies and patient-centred care tailored to the specific needs of this population. The objectives of this study are: (1) to characterise the OHRQoL in a Portuguese paediatric population with DM1; (2) to compare oral health behaviours, perceptions of oral health, and reported oral manifestations between individuals with DM1 and those without the disease; (3) to analyse the factors associated with OHRQoL in both groups (with and without DM1).

## 2. Materials and Methods

An observational, comparative, and cross-sectional study was conducted, authorised by the Health Ethics Committee of Faculdade de Medicina Dentária da Universidade de Lisboa (ref: CE-FMDUL 202438).

The target population consisted of children and adolescents under 18 years of age who are Portuguese and diagnosed with DM1. For comparison, individuals of the same age group, Portuguese, and without DM were also included.

The questionnaire was made available online and disseminated nationwide in Portugal. The sample was non-probabilistic and included all participants under 18 whose parents voluntarily completed the online questionnaire and provided consent for their participation in the study. To stratify the sample by age group, the questionnaire was initially distributed to parents or guardians of individuals with DM1. After collecting their responses, they were matched with those of participants without the disease based on their age groups (0–5 years, 6–11 years, and 12–17 years). Considering the proportion of DM1 participants in these age groups, the questionnaire was then provided to parents or guardians of children or adolescents without the disease. Once a similar proportion was reached for each age group, the questionnaire was closed for that group.

Data collection was conducted using an online questionnaire completed by the parents themselves. This questionnaire was previously developed and used in a cross-sectional study involving a Portuguese paediatric population (less than 18 years old) with celiac disease [15]. It was adapted for individuals with DM1, with a particular focus on questions related to the disease and its oral manifestations. These adaptations were based on a literature review [16,17,18,19]. The questionnaire was administered to both groups (with and without DM1), with the only difference being the inclusion of questions about DM, which were not present in the questionnaire for children without the disease.

The questionnaire was reviewed in advance by a panel of experts with experience in oral health and questionnaire research. A pilot test was also conducted with two participants, parents of children with DM1, whose responses were excluded from the study sample. These steps helped identify difficulties in understanding the questionnaire and errors in its computer design, leading to minor adjustments to enhance clarity, usability, and data reliability.

The final questionnaire introduced the study and its objectives on the first page, noting that participation was entirely voluntary and anonymous. The initial questions addressed informed consent and adherence to the criteria of the target population; therefore, participants proceeded with the questionnaire only if they agreed to participate and met these criteria. The questionnaire itself listed all questions as mandatory, and consequently, no missing data were generated. It collected information on sociodemographic characteristics, self-reported diabetes control, oral health behaviours, perceptions of oral health, self-reported oral manifestations, and the Portuguese version of the “Early Childhood Oral Health Impact Scale” (ECOHIS, Table S1) [20].

The ECOHIS is a tool originally developed to assess OHRQoL in children aged 0–6 years. It comprises thirteen items, nine of which evaluate the impact of oral health issues on the child (ECOHIS Child), while four focus on the effect on the family (ECOHIS Family). All years in the child’s life, from birth to the present, are considered within this scale. The score for each participant depends on their responses to each item, with options: “Never” = 0, “Almost never” = 1, “Occasionally” = 2, “Often” = 3, and “Very often” = 4. The option “I don’t know” is the sixth choice and is regarded as a “missing value”. Participants with more than two missing answers for items related to the child, or more than one for items pertaining to the family, were excluded from the study. However, for questionnaires with up to two “I don’t know” answers in the child’s section or one in the family section, the value was assigned based on the average of responses within that same subscale. The ECOHIS total score is calculated by summing all item values, ranging from a minimum of “0” to a maximum of “52”. A lower score indicates a lesser impact of OHRQoL on the child and their family [21]. This scale has already been validated for the Portuguese population in studies with adolescents [20] and preschoolers [22]. ECOHIS has robust internal consistency, test–retest reliability, discriminant validity, and documented responsiveness to clinical change across multiple languages, facilitating comparisons. The Portuguese version demonstrates good psychometrics, including in paediatric populations spanning adolescent ages [20], and has been applied in another Portuguese study involving a diverse paediatric age group [15]. The ECOHIS was also used in a mixed paediatric multicentre study, which used a cohort of children and adolescents with juvenile idiopathic arthritis. Investigators applied and examined the validity and internal consistency of ECOHIS (alongside Child-OIDP), indicating feasibility and acceptable measurement properties beyond preschool ages [23].

To distribute the questionnaire within the DM1 group, several Portuguese associations related to the disease were contacted, and the study protocol was shared with them. However, despite numerous attempts to reach each other via email and telephone, only one association responded positively to disseminate the questionnaire. Due to this difficulty, it was decided to share the questionnaire link on social networks, specifically in discussion and sharing groups dedicated to DM1. Even so, participation was lower than expected, and parents enrolled in these discussion groups were contacted individually through social networks to increase the number of responses. The questionnaire was also shared in groups without DM1 on social networks, including parent discussion groups, parent associations, and school groups. Data collection took place between March and May 2025.

Statistical analysis included calculating absolute and relative frequencies, as well as the mean and standard deviation for numerical variables. Pearson’s chi-square test was used to compare the groups (with and without DM1) regarding sociodemographic, oral manifestations, and behavioural variables. The ECOHIS total scores of the groups were compared using the non-parametric Mann–Whitney U test.

Due to the non-normal distribution of the outcome variable and evidence of overdispersion (variance significantly exceeding the mean), a negative binomial regression model was used to explore factors associated with the OHRQoL (ECOHIS total) within each group. Model fit was assessed by examining the Pearson chi-square and deviance statistics, both of which indicated an adequate fit. Variables included in the negative binomial regression models were selected based on an initial exploratory bivariate analysis using non-parametric tests, specifically the Mann–Whitney and Kruskal–Wallis tests. The first multivariate model incorporated all variables with a *p*-value ≤ 0.25 in the bivariate analysis. A manual stepwise procedure was then performed to remove variables with a *p*-value > 0.10, starting with the least significant. The exclusion of predictors was also used as a criterion for evaluating the model’s fit. The mother’s level of education was retained in the final model regardless of statistical significance, due to its well-known role as a social determinant of children’s oral health. All tests used a significance level of 5%.

## 3. Results

The sample comprised 235 participants, including 120 parents or guardians of individuals without DM1 and 115 parents or guardians of individuals with DM1.

The characterisation of the sample is detailed in Table 1. The age group with the most responses was 12–17 years old, with nearly 50% of the responses in both groups. The child’s sex was statistically significant (*p* = 0.01), with a higher proportion of males in the non-diabetic group (Table 1).

Most individuals (83.5%) reported managing DM effectively, and only 7.8% indicated the presence of an additional condition that could impact oral health (Table 1). These additional conditions included celiac disease and asthma. Regarding the age at diagnosis of DM, the majority were diagnosed at or below seven years of age (66.1%), with an average age of 6.4 years (SD = 3.8) and a range from 1 to 15 years.

### 3.1. Oral Health-Related Quality of Life

The frequencies and means of each ECOHIS item for the DM1 and non-DM1 groups are detailed in Table 2 and Table 3, respectively. In both groups, it was observed that most participants marked the hypothesis “Never” on the majority of the ECOHIS questions.

Among DM1 children, according to parents’ or guardians’ perceptions, symptoms were the aspect exerting the most significant impact on the child’s OHRQoL, as 42.6% reported that the child or adolescent had experienced tooth or mouth pain. Conversely, the domains of self-image and social interaction proved to be the least impactful on the child’s OHRQoL, with over 86% of responses indicating ‘Never’. Regarding the family subscale, it was observed that 43.9% of the parents/guardians reported a financial impact on the family caused by the costs associated with their child’s oral health problems. These problems also affected their well-being, as 27.9% stated that they had already been upset due to their child’s dental problems (Table 2).

Similarly, in the group without DM1, symptoms were the factor with the most significant impact on the child’s OHRQoL, as 40% of the children or adolescents had already experienced a toothache or mouth pain. The self-image and social interaction domain, similar to the DM1 group, showed the least impact on the child’s OHRQoL, with over 85% of responses being “Never”. Regarding the family subscale, the most significant impact was attributed to costs related to oral health issues in children and adolescents, with 40.8% of parents or guardians reporting that dental problems or treatments influenced their family budget and work attendance. Additionally, 27.5% reported taking time off from work (Table 3).

The ECOHIS child score was similar in both groups, with a mean of 2.39 (SD = 3.39) in children with DM1 and 2.39 (SD = 3.28) in children without the disease. In contrast, the family subscale score was slightly higher in individuals with DM1 (2.16) compared to those without it (1.63). The ECOHIS total mean value for children with DM1 was also slightly higher (4.38 vs. 4.02), with a minimum value of “0” in both groups and a maximum of “34” and “24” for children with and without DM1, respectively. None of the values showed significant differences between the two groups (*p* > 0.05) (Table 4).

### 3.2. Oral Health Behaviours and Perception

Regarding oral health behaviours, most caregivers reported that children and adolescents brushed their teeth at least twice daily, with a lower percentage observed in individuals with diabetes (70.4%) compared to those without diabetes (75.8%), but with no significant differences (*p* > 0.05). Analysing how often individuals consume sugary foods and drinks, the most common response in both groups was “a few times a week (2–3 times)”, accounting for 37.4% of the DM1 group and 54.2% of the non-DM1 group. The tendency of DM1 children to consume less of this type of food was not statistically significant (*p* = 0.08) (Table 5).

In both groups, the frequency of regular oral health appointments (1–2 times a year) was high (Table 5); however, only 10.4% of parents or guardians of children with diabetes reported receiving specific recommendations on oral health care, namely “extra reinforcement in oral hygiene care”, “brush your teeth or at least rinse after a hypoglycaemic episode in which it is necessary to consume sugar”, and “be especially careful in eating, preventing it from being cariogenic, not only because of diabetes, but also because of the risk of developing dental caries”.

The perception of oral health varied significantly between the groups (*p* < 0.001), with parents of children with DM1 rating their children’s oral health more negatively. Despite these differences, most of them reported that their children’s oral health was “very good” or “good” (Table 5).

### 3.3. Oral Manifestations Reported

The most frequently reported oral symptoms were dental caries (49.6% in DM1 and 54.2% in non-DM1) and halitosis (32.2% in DM1 and 26.7% in non-DM1) (*p* > 0.05). Among individuals with DM1, 20% of parents reported experiencing dry mouth, compared to only 5.8% of parents of those without DM1 (*p* = 0.001) (Table 6).

More than two-thirds of parents and guardians (65.2%) report improvements in their child’s oral health when diabetes is controlled. Meanwhile, 9.6% state that the child or adolescent has not yet gained control over the condition.

### 3.4. Factors Associated with Oral Health-Related Quality of Life (ECOHIS Total)

The final negative binomial regression model of the children with diabetes identified several factors significantly associated with better oral health-related quality of life (lower ECOHIS total scores). Children without reported dental caries (β = −0.89, *p* < 0.001), gingivitis (β = −1.19, *p* < 0.001), or changes in taste (β = −0.94, *p* = 0.019), and those who were female (β = −0.66, *p* = 0.003), had significantly lower ECOHIS scores, demonstrating a better OHRQoL. Although not statistically significant (β = −0.31, *p* = 0.200), maternal education level was retained in the final model because of its recognised importance as a social determinant of children’s oral health (Table 7).

This final binomial regression model for children with DM1 demonstrated a good fit to the data, with a deviance-to-degrees-of-freedom ratio of 1.291 and a Pearson chi-square-to-degrees-of-freedom ratio of 1.053. Information criteria further supported the adequacy of the model, with AIC = 549.828 and BIC = 566.192. This model was selected after testing several alternatives that exhibited poorer fit.

The final negative binomial regression model for children without DM1 is presented in Table 8. Children in the youngest age groups had significantly lower ECOHIS total scores compared to the oldest group (β = −1.26, *p* = 0.001 for 0–5 years old; and β = −1.06, *p* < 0.001 for the 6–11 years old), indicating better OHRQoL. Children who did not frequently consume sugary foods (β = −1.11, *p* < 0.001) and those with no history of recurrent mouth ulcers (β = −0.75, *p* = 0.011) or burning sensations in the mouth (β = −1.32, *p* = 0.008) also had significantly better oral health-related quality of life. Caregivers’ poorer perceptions of the child’s oral health were associated with higher ECOHIS total scores, particularly for those rated as “poor” (β = 0.93, *p* < 0.001) and “reasonable” (β = 1.06, *p* = 0.018). The mother’s education level was retained in the final model due to its theoretical importance, despite being non-significant (*p* = 0.53), as was the case in the model of the DM1 group.

This final negative binomial regression model for the children without DM1 also demonstrated an adequate fit, with a Pearson chi-square to degrees of freedom ratio of 1.15 and a deviance-to-degrees-of-freedom ratio of 1.16, both of which are close to 1, indicating good model specification. The model also showed a lower AIC (564.30) and improved log-likelihood (−272.15) when compared to earlier exploratory models tested during the analysis, supporting its relative superiority in explaining the variability in ECOHIS total.

## 4. Discussion

The main objective of this study was to assess oral health-related quality of life (OHRQoL) in a Portuguese paediatric population with DM1 and to compare it with that of a matched non-DM1 control group. Using the Portuguese version of the ECOHIS instrument, validated for both preschool and adolescent populations, this study aimed to characterise the impact of diabetes on the functional, emotional, social, and family dimensions of children’s lives. While overall OHRQoL scores were favourable, these findings do not eliminate the need for oral health promotion strategies specifically tailored to the needs and risks of this clinical population.

The ECOHIS values in this study are relatively low, suggesting a good OHRQoL among children with DM1. In fact, low ECOHIS scores have been noted in numerous studies involving the paediatric population overall, as well as in groups with specific health conditions. However, even in these studies where the overall scores are satisfactory, many oral diseases are reported to have a considerable negative impact on OHRQoL [15,24,25,26,27].

In the group with DM1, the symptoms domain was the most impactful, with many parents (42.6%) reporting that the child/adolescent has already felt orofacial pain. This data aligns with other cross-sectional studies conducted in Portuguese populations, which indicate that this domain has the most significant impact [15,24]. One of these studies made in a paediatric population with celiac disease revealed that 53.6% of the parents reported orofacial pain [15]. The other study had a lower value (23.3%), but it was made in preschool ages [24]. The social domain was not as impactful, contrary to what was observed in a study of Egyptian adolescents with DM1 [28]. These differences in results may be related to the scale used, as the ECOHIS is a scale that relies solely on parents’ responses, and caregivers’ perceptions may be more sensitive to visible or reported physical manifestations, such as pain, than to emotional or social changes. In fact, the domain with the least impact was self-esteem/social interaction, with more than 85% of the responses indicating “never” for issues such as avoiding laughing or talking. Since the answers are provided by the parents and not by the children or adolescents themselves, this can explain the differences [29]. On the other hand, this study did not include only adolescents, and in children at younger ages, social conditions are usually less impactful [22,24].

In the family subscale, the financial impact of dental treatments was significant (43.9%), indicating that the economic burden of oral health on families with children with DM1 should be considered when developing public policies.

The ECOHIS total for the DM1 group was 4.38, slightly higher than in the group without DM1 (4.02), although this difference was not statistically significant. However, maximum values were higher in the diabetic group (34 vs. 24), indicating that specific subgroups within the DM1 population may be more vulnerable to this condition compared to non-DM1 individuals. These findings suggest that health professionals, and particularly oral health professionals, should be particularly vigilant and aware of patients with DM1 and the impact of oral diseases on their well-being, directing targeted efforts towards preventing oral diseases.

No statistically significant differences were observed in total ECOHIS between the two groups (*p* > 0.05). These results do not align with a study conducted in a preschool population with DM1, which found significant differences in the ECOHIS family [30]. In the present study, the absence of differences between the groups may be due to well-controlled diabetes in 83.5% of diabetic participants, as disease management is recognised as a key factor influencing the development of oral manifestations that can affect quality of life [1]. Nonetheless, some studies suggest that DM1 has no statistically significant association with OHRQoL [11,12].

Both groups generally displayed good oral hygiene behaviours, with approximately 70–75% of children brushing their teeth at least twice daily and over 90% attending regular oral health appointments. Interestingly, children with DM1 brushed their teeth slightly less frequently (70.4% vs. 75.8%), although this difference was not statistically significant (*p* = 0.20). This finding warrants attention, as consistent twice-daily toothbrushing is not only crucial for preventing caries and periodontal inflammation, but it also appears to be linked to improved glycaemic control in individuals with diabetes. A recent scoping review suggests that improved oral hygiene behaviours, including toothbrushing frequency, may help reduce HbA1c levels [31].

On the other hand, children with DM1 consumed sugary foods and drinks less frequently, reflecting more controlled eating habits. This is an expected pattern in individuals who are routinely advised to restrict simple carbohydrate intake as part of their DM1 management plan. Although this difference did not reach statistical significance (*p* = 0.08), it trends towards significance and aligns with clinical recommendations for the metabolic control of DM1. The improvement of dietary habits appears to be important for enhancing OHRQoL in both children with and without diabetes. In this context, a recent study demonstrated that increasing parents’ nutritional literacy can be a key factor in promoting healthier eating behaviours, thereby contributing to better OHRQoL [32].

Parents’ perception of oral health was significantly more negative in the DM1 group (*p* < 0.001), despite reporting good behaviours. This may reflect greater awareness of risks or previous negative experiences; however, in regression models, this variable was only associated with the ECOHIS total in the non-DM1 group. This finding might suggest some adjustment among caregivers of children with diabetes, who may regard oral symptoms as a regular part of DM1 management and therefore underestimate their impact on daily life. Conversely, in children without DM1, such symptoms may be more noticeable as deviations from expected oral health, leading to a stronger connection with perceived quality of life. This relationship is supported by previous studies, which identify perceived oral health as a significant predictor of OHRQoL, given its role in capturing the child’s or caregiver’s subjective experience of oral health and its influence on well-being [15,24].

The most commonly reported oral manifestations were dental caries and halitosis in both groups. However, xerostomia was significantly more prevalent in children with DM1 (20.0%) compared to those without the disease (5.8%) (*p* = 0.001), supporting the described relationship between DM1 and reduced salivary flow [1,6,33].

The regression models revealed distinct patterns of association with OHRQoL between children with and without DM1, emphasising the need for tailored approaches in oral health promotion.

Among children with DM1, better OHRQoL was significantly associated with the absence of oral conditions, including dental caries, gingivitis, and changes in taste. These findings reinforce the burden of these symptoms on daily functioning and comfort [28]. Additionally, being female was associated with a better OHRQoL in this group. While some research reports worse outcomes among girls with poor glycaemic control [34], this result may reflect behavioural or perceptual differences in symptom awareness, self-care, or family support structures. However, this difference between the sexes is not consensual, and the influence of sex on OHRQoL remains complex and context-dependent.

Interestingly, although caregivers of children with DM1 tended to rate their child’s oral health more negatively, this perception was not significantly related to OHRQoL in this group. One possible reason is the normalisation of oral symptoms within the context of a chronic disease. Caregivers may view problems such as dryness, discomfort, or mild infections as expected aspects of diabetes management, thereby underestimating their particular impact on daily life. In the group without DM1, caregivers’ negative perceptions of oral health were clearly associated with worse OHRQoL scores, possibly reflecting increased sensitivity to oral changes in otherwise healthy children. In the absence of an underlying systemic condition, such symptoms may be more disruptive or unexpected, with a greater perceived impact. The association between poorer oral health perception and a worse OHRQoL found in the children without DM1 has also been found in other studies [15,22,24,35].

Other factors that impacted OHRQoL among children without DM1 included older age, frequent consumption of sugary foods, and symptoms such as recurrent aphthous stomatitis and burning mouth sensations. Burning sensations and aphthous stomatitis are typically oral health problems less discussed in healthy populations, but were significantly associated with the worst OHRQoL, supporting a broader understanding of these symptoms beyond traditional dental indicators. Behavioural factors, such as frequent intake of sugary foods, were associated with worse OHRQoL, consistent with findings from Libya, where dietary habits and oral health behaviours are strongly linked to perceived quality of life [36]. Age is also a well-defined factor related to the OHQoL in children [37,38,39], as confirmed in this study.

Overall, these findings support the importance of recognising the distinct oral health profiles and needs of children with DM1, and of integrating oral health more systematically into chronic disease management to promote overall health and quality of life. OHRQoL has a multifactorial nature, requiring differentiated and context-sensitive approaches. In children with chronic systemic conditions, such as DM1, oral health professionals must be aware of their role in supporting glycaemic control and overall health in these children. Given the bidirectional relationship between periodontal inflammation and diabetes, tailored oral health advice is critical in this population [31,40]. However, only a small proportion of caregivers reported having received such guidance, highlighting a gap in dental care. Specific recommendations for diabetic children should include an increased frequency of dental routines, early detection of oral manifestations, and reinforcement of dietary counselling that aligns with both caries prevention and glycaemic control. Strengthening the involvement of dental professionals in multidisciplinary diabetes care is important to ensure that oral health guidance is both systematic and condition-specific.

Although this study has inherent limitations due to its cross-sectional design and reliance on parent-reported oral health conditions, its findings cannot be extrapolated to the entire paediatric DM1 population; nonetheless, it offers a valuable contribution. The non-probabilistic sample can introduce some selection bias, such as socioeconomic status or access to dental care. Age-group matching was performed between groups; however, it was not possible to match for gender or socioeconomic status. Furthermore, the oral health outcomes reported were not clinically confirmed. These potential confounding factors must be taken into consideration. Additionally, the sample consisted of only 115 children and adolescents with DM1. Still, despite the reduced sample size, it accounted for approximately 3.8% of the estimated 2982 individuals aged 19 or younger with DM1 in Portugal, which represents a meaningful proportion for an exploratory analysis. Nevertheless, despite these limitations, this study offers an integrated view by combining reported diseases, symptom-related variables, and behavioural factors, providing a comprehensive analysis of OHRQoL outcomes. Furthermore, the observed data can be useful in enabling the adoption of preventive oral health strategies in a clinical setting, as well as informing public health policies, as they emphasise the need for greater targeted attention to the specific care needs of this susceptible population.

## 5. Conclusions

Based on the results of this study, it can be concluded that:

- Although no statistically significant differences were found in overall OHRQoL between children with and without DM1, the factors associated with OHRQoL differed substantially between the two groups, highlighting the importance of tailored oral health strategies.
- In children with DM1, better OHRQoL was mainly associated with the absence of oral conditions such as caries, gingivitis, and taste changes, as well as with female sex. In contrast, among children without DM1, OHRQoL was more strongly influenced by caregiver perception of oral health, the presence of symptoms such as recurrent aphthous stomatitis and burning mouth sensations, frequent sugar intake, and older age.
- Both groups reported generally adequate oral health behaviours, although only a minority of caregivers of children with DM1 indicated having received specific oral health guidance from professionals.

## Figures and Tables

**Table 1:** Characterisation of sample based on sociodemographic information and presence of additional pathology.

Variable	Children with DM1% (*n*)	Children Without DM1% (*n*)	*p*-Value *
**Age**			
0–5 years old	13.0% (15)	13.3% (16)	0.95
6–11 years old	37.4% (43)	39.2% (47)	
12–17 years old	49.6% (57)	47.5% (57)	
**Sex**			
Female	43.5% (50)	40.0% (48)	**0.01**
Male	56.5% (65)	60.0% (72)	
**Mother’s education level**			
Less than basic education	3.5% (4)	9.2% (11)	0.22
Basic education (9 years)	7.8% (9)	10.8% (13)	
Secondary education (12 years)	33.0% (38)	33.3% (40)	
Higher education	55.7% (64)	46.7% (56)	
**Presence of additional pathology**			
No	92.2% (106)	95.8% (115)	0.24
Yes	7.8% (9)	4.2% (5)	

* Pearson’s chi-square test; *p*-values in bold are statistically significant.

**Table 2:** Frequencies and means of ECOHIS items in DM1 group.

Domain	Item	Never	Hardly Ever	Occasionally	Often	Very Often	Mean (sd)
		% (*n*)	% (*n*)	% (*n*)	% (*n*)	% (*n*)	
Child symptoms	Pain in the teeth, mouth or jaws	57.4 (66)	28.7 (33)	11.3 (13)	2.6 (3)	0.0 (0)	0.59 (0.79)
Child Function	Difficulty drinking hot or cold beverages	71.3 (82)	21.7 (25)	4.3 (5)	2.6 (3)	0.0 (0)	0.38 (0.70)
	Difficulty eating some foods	72.8 (83)	24.6 (28)	2.6 (3)	0.0 (0)	0.0 (0)	0.30 (0.51)
	Difficulty pronouncing any words	85.2 (98)	11.4 (13)	2.6 (3)	0.0 (0)	0.0 (0)	0.17 (0.44)
	Missed school	81.6 (93)	15.8 (18)	1.8 (2)	0.9 (1)	0.0 (0)	0.22 (0.51)
Child psychological	Trouble sleeping	80.0 (92)	15.7 (18)	1.7 (2)	2.6 (3)	0.0 (0)	0.27 (0.63)
	Been irritable or frustrated	78.3 (90)	15.7 (18)	5.2 (6)	0.0 (0)	0.9 (1)	0.30 (0.65)
Child self-image/social interaction	Avoided smiling or laughing	86.1 (99)	10.4 (12)	2.6 (3)	0.0 (0)	0.9 (1)	0.19 (0.56)
	Avoided talking	89.6 (103)	7.8 (9)	1.7 (2)	0.0 (0)	0.9 (1)	0.15 (0.52)
Parent distress	Been upset	72.1 (83)	14.8 (17)	11.3 (13)	0.9 (1)	0.9 (1)	0.43 (0.8)
	Felt guilty	75.7 (87)	11.3 (13)	9.6 (11)	2.6 (3)	0.9 (1)	0.42 (0.84)
Family function	Taken time off from work	72.8 (83)	18.4 (21)	7.0 (8)	0.0 (0)	1.8 (2)	0.39 (0.77)
	Financial impact	56.1 (64)	16.7 (19)	13.2 (15)	8.8 (10)	5.3 (6)	0.90 (1.23)

**Table 3:** Frequencies and means of ECOHIS items in non-DM1 group.

Domain	Item	Never	Hardly Ever	Occasionally	Often	Very Often	Mean (sd)
		% (*n*)	% (*n*)	% (*n*)	% (*n*)	% (*n*)	
Child symptoms	Pain in the teeth, mouth or jaws	60.0 (72)	31.7 (38)	7.5 (9)	0.8 (1)	0.0 (0)	0.49 (0.67)
Child Function	Difficulty drinking hot or cold beverages	69.2 (83)	26.7 (32)	4.2 (5)	0.0 (0)	0.0 (0)	0.35 (0.56)
	Difficulty eating some foods	70.8 (85)	23.3 (28)	5.8 (7)	0.0 (0)	0.0 (0)	0.35 (0.59)
	Difficulty pronouncing any words	83.3 (100)	10.8 (13)	5.8 (7)	0.0 (0)	0.0 (0)	0.23 (0.54)
	Missed school	85.8 (103)	12.5 (15)	0.8 (1)	0.8 (1)	0.0 (0)	0.17 (0.46)
Child psychological	Trouble sleeping	82.5 (99)	13.3 (16)	3.3 (4)	0.8 (1)	0.0 (0)	0.23 (0.54)
	Been irritable or frustrated	79.2 (95)	16.7 (20)	3.3 (4)	0.8 (1)	0.0 (0)	0.26 (0.56)
Child self-image/social interaction	Avoided smiling or laughing	85.8 (103)	10.8 (13)	1.7 (2)	0.8 (1)	0.8 (1)	0.2 (0.59)
	Avoided talking	90.0 (108)	8.3 (10)	0.8 (1)	0.8 (1)	0.0 (0)	0.13 (0.42)
Parent distress	Been upset	77.5 (93)	19.2 (23)	3.3 (4)	0.0 (0)	0.0 (0)	0.26 (0.51)
	Felt guilty	81.7 (98)	11.7 (14)	6.7 (8)	0.0 (0)	0.0 (0)	0.25 (0.57)
Family function	Taken time off from work	72.5 (87)	16.7 (20)	8.3 (10)	1.7 (2)	0.8 (1)	0.42 (0.78)
	Financial impact	59.2 (71)	22.5 (27)	10.0 (12)	5.8 (7)	2.5 (3)	0.70 (1.03)

**Table 4:** Comparison of ECOHIS between children with and without DM1.

	Children with DM1	Children Without DM1	*p*-Value *
	Mean (sd)	Mean (sd)	
ECOHIS child	2.39 (3.39)	2.39 (3.28)	0.93
ECOHIS family	2.16 (3.08)	1.63 (2.35)	0.36
ECOHIS total	4.38 (5.63)	4.02 (5.06)	0.80

* Mann–Whitney U test.

**Table 5:** Comparison of oral health behaviours and perception between children with and without DM1.

Variable	Children with DM1% (*n*)	Children Without DM1% (*n*)	*p*-Value *
**Toothbrushing frequency**			
Doesn’t brush because doesn’t have teeth	0.0% (0)	0.8% (1)	0.20
Less than once a day	2.6% (3)	0.0% (0)	
Once a day	27.0% (31)	23.3% (28)	
Twice or more per day	70.4% (81)	75.8% (91)	
**Sugary foods intake frequency**			
Never or rarely	25.2% (29)	18.3% (22)	0.08
Sometimes a week (2–3 times)	37.4% (43)	54.2% (65)	
Many times a week (more than 3 times)	24.3% (28)	17.5% (21)	
Every day	13.0% (15)	10.0% (12)	
**Oral health appointments at least once/year**			
Yes	93.0% (107)	90.0% (108)	0.40
No	7.0% (8)	10.0% (12)	
**Oral health perception**			
Very good	24.3% (28)	35.8% (43)	**<0.001**
Good	46.1% (53)	55.8% (67)	
Reasonable	27.0% (31)	7.5% (9)	
Bad	2.6% (3)	0.8% (1)	
Very bad	0.0% (0)	0.0%(0)	

* Pearson’s chi-square test; *p*-values in bold are statistically significant.

**Table 6:** Comparison of oral manifestations reported between children with and without DM1.

Oral Manifestations Reported	Children with DM1% (*n*)	Children Without DM1% (*n*)	*p*-Value *
Dental caries	49.6% (57)	54.2% (65)	0.48
Recurrent canker sores	16.5% (19)	18.3% (22)	0.72
Gingivitis	9.6% (11)	8.3% (10)	0.74
Xerostomia	20.0% (23)	5.8% (7)	**0.001**
Inflammation or lesions in the mucosa	12.2% (14)	11.7% (14)	0.90
Halitosis	32.2% (37)	26.7% (32)	0.35
Burning in the mouth	3.5% (4)	4.2% (5)	0.78
Taste changes	7.8% (9)	5.0% (6)	0.37

* Pearson’s chi-square test; *p*-values in bold are statistically significant.

**Table 7:** Factors associated with OHRQoL (ECOHIS total) in children with DM1.

	β	Standard Error	*p*-Value *	95% Confidence Interval	
				Lower Limit	Upper Limit
Intercept	4.02	0.57	**<0.001**	2.91	5.13
Female	−0.66	0.23	**0.003**	−0.10	−0.22
Mother without higher education	−0.31	0.24	0.20	−0.78	0.16
No report of dental caries	−0.89	0.23	**<0.001**	−1.35	−0.43
No report of gingivitis	−1.19	0.35	**<0.001**	−1.88	−0.50
No report of changes in taste	−0.94	0.40	**0.019**	−1.72	−0.16

* Negative binomial regression; *p*-values in bold are statistically significant.

**Table 8:** Factors associated with OHRQoL (ECOHIS total) in children without DM1.

	β	Standard Error	*p*-Value *	95% Confidence Interval	
				Lower Limit	Upper Limit
Intercept	3.20	0.59	**<0.001**	2.04	4.34
0–5 years old	−1.26	0.39	**0.001**	−2.01	−0.50
6–11 years old	−1.06	0.26	**<0.001**	−1.58	−0.54
Mother without higher education	−0.15	0.24	0.53	−0.62	0.32
Frequent intake of sugary foods	−1.11	0.33	**<0.001**	−1.75	0.47
Bad oral health perception	2.10	1.15	0.07	−0.18	4.33
Reasonable oral health perception	1.06	0.45	**0.018**	0.18	1.93
Good oral health perception	0.93	0.26	**<0.001**	0.43	1.44
No report of recurrent aphthous stomatitis	−0.75	0.29	**0.01**	−1.33	−0.18
No report of oral burning	−1.32	0.50	**0.008**	−2.31	−0.34

* Negative binomial regression; *p*-values in bold are statistically significant.

## Supplementary Materials

The following supporting information can be downloaded at: https://www.mdpi.com/article/10.3390/dj13090425/s1, Table S1: ECOHIS—Portuguese version (Portugal).

## Abbreviations

The following abbreviations are used in this manuscript:

DM1	Type 1 Diabetes
OHRQoL	Oral Health-Related Quality of Life
SD	Standard deviation
