# Supplementary material for: Oral Health-Related Quality of Life, Behaviours and Oral Manifestations in a Paediatric Population with Type I Diabetes Mellitus: A Comparative Cross-Sectional Study
Source: Dent J (Basel). 2025 Sep 15;13(9):425. doi: 10.3390/dj13090425 (PMC12468200; doi:10.3390/dj13090425)
Supplement: Supplementary file 1 [file dentistry-13-00425-s001.zip › dentistry-3809807-SI.pdf]

ECOHIS – Portuguese version (Portugal)

Para responder às questões que se seguem, considere a vida completa da criança desde o nascimento até ao momento presente. Coloque uma única cruz no quadrado correspondente à situação que melhor descreve a sua experiência.
Se a pergunta não se aplica, marque "Nunca".

	**Nunca**	**Quase nunca**	**Ocasional Mente**	**Frequente mente**	**Muito frequente mente**	**Não sei**
Com que frequência o seu filho(a)/educando teve dor de dentes/boca?						
Com que frequência o seu filho(a)/educando teve dificuldade em ingerir bebidas quentes ou frias devido a problemas dos dentes ou com o tratamento dos dentes?						
Com que frequência o seu filho(a)/educando teve dificuldade em ingerir algum(s) alimento(s) devido a problemas dos dentes ou com o tratamento dos dentes?						
Com que frequência o seu filho(a)/educando teve dificuldade em pronunciar alguma(s) palavra(s) devido a problemas dos dentes ou com o tratamento dos dentes?						
Com que frequência o seu filho(a)/educando faltou à creche, infantário ou escola devido a problemas dos dentes ou com tratamento dos dentes?						
Com que frequência o seu filho(a)/educando teve problemas em adormecer devido a problemas com os dentes ou tratamento dos dentes?						
Com que frequência o seu filho(a)/educando esteve frustrado devido a problemas com os dentes ou com o tratamento dos dentes?						
Com que frequência o seu filho(a)/educando evitou sorrir ou rir em frente a outras crianças devido a problemas com os dentes ou com tratamento dos dentes?						
Com que frequência o seu filho(a)/educando evitou falar com outras crianças devido a problemas com os dentes ou com o tratamento dos dentes?						
Com que frequência você ou outro membro da família esteve aborrecida(o)/chateada(o) devido a problemas com os dentes ou tratamento dos dentes do seu filho(a)/educando?						
Com que frequência você ou outro membro da família se sentiu culpada(o) devido a problemas com os dentes ou tratamento dos dentes do seu filho(a)/educando?						
Com que frequência você ou outro membro da família faltou ao trabalho devido a problemas com os dentes ou tratamento dos dentes do seu filho(a)/educando?						
Com que frequência os problemas com os dentes do seu filho(a)/educando e os seus tratamentos tiveram impacto financeiro na sua família?						
